# Urinary volatilome analysis in a mouse model of anxiety and depression

**DOI:** 10.1371/journal.pone.0229269

**Published:** 2020-02-21

**Authors:** Akiko Fujita, Takaya Okuno, Mika Oda, Keiko Kato

**Affiliations:** Faculty of Life Sciences, Kyoto Sangyo University, Kyoto, Japan; Duke University, UNITED STATES

## Abstract

Psychiatric disorders including depression and anxiety comprise a broad range of conditions with different symptoms. We have developed a mouse model of depression/anxiety in mice deficient in the *St3gal4* gene. In this study, we performed a comparative analysis of urinary volatile organic compounds (VOCs) in *St3gal4*-deficient (St3gal4-KO) and wild-type mice using gas chromatography-mass spectrometry, and we screened 18 putative VOCs. Principal component analysis (PCA) based on these VOCs identified a major group of 11 VOCs, from which two groups were clarified by hierarchical clustering analysis. One group including six VOCs (pentanoic acid, 4-methyl-, ethyl ester; 3-heptanone, 6-methyl; benzaldehyde; 5,9-undecadien-2-ol, 6,10-dimethyl; and unknown compounds RI1291 and RI1237) was correlated with the startle response (*r* = 0.620), which is related to an unconscious defensive response. The other group including two VOCs (beta-farnesene and alpha-farnesene) comprised pheromones which increased in KO mice. Next, male mice underwent a social behavior test with female mice in the estrus stage, showing reduced access of KO male mice to female mice. Comparative analysis of urinary VOCs before and after encounters revealed that the six VOCs were not changed by these encounters. However, in WT mice, the two farnesenes increased after the encounters, reaching the level observed in KO mice, which was not altered following the encounter. Taken together, these results indicated that St3gal4 was involved in modulating urinary VOCs. Moreover, VOC clusters discovered by comparison of St3gal4-KO mice with WT mice were correlated with differential emotional behaviors.

## Introduction

Depression and anxiety are common mental disorders that co-occur frequently [1], and they have been identified as a leading cause of burden globally [2]. Depression is a psychiatric disorder that is characterized by a depressed mood and/or loss of interest or pleasure and causes severe symptoms that affect daily activities, such as sleeping, eating, or working. Anxiety is characterized by feelings of anxiety and fear [3]. In order to help patients who suffer from mental illness, it is important to deepen our understanding of the molecular mechanisms underlying psychiatric disorders and to investigate the potential mechanisms of action of drugs in patients with these disorders. Animal models, particularly rodent models, are valuable tools in this regard [4].

We previously reported that ST3 beta-galactoside alpha-2,3-sialyltransferase IV (*St3gal4*)-deficient mice showed symptoms of anxiety and depression [5]. St3gal4 is one of the six sialyltransferases (St3gal1–6) that generate Sia-2,3Gal linkages on the ends of glycoproteins. The correlation between enzymes involved in synthesis of sialoglycan and psychiatric disorders have been reported in humans. In humans, the GM3 synthase *ST3GAL5* is associated with severe infantile-onset seizures, developmental delay, and blindness [6–8]. Moreover, mutations in *ST3GAL3* result in nonsyndromic intellectual disability [9] and West syndrome [10]. Genome-wide association studies have demonstrated associations between *ST3GAL4* gene variants or single-nucleotide polymorphisms and lipid traits, including high ApoB levels, total cholesterol, triglyceride levels, and coronary artery disease [11,12]. In mouse, St3gal4 expression is region specific, showing abundant expression in the thalamic sensory relay nuclei, including the medial and lateral geniculate, inter geniculate, and ventroposterior nuclei [5,13]. Additionally, St3gal4 is upregulated in neurons located within the neural circuits that exhibit propagation of kindling stimulation [13]. In contrast, St3gal4-knockout (KO) mice exhibit no epileptic seizures [5]. Furthermore, emotional behavioral tests, including open-field tests, forced swim tests, and fear conditioning tests, showed depression- and anxiety-like symptoms in these mice [5]. We found that kindling stimulation increased growth hormone (GH) levels in neuronal cells and upregulated St3gal4 [14]. In contrast, St3gal4-KO mice showed downregulation of *GH* and insulin-like growth factor 1 (*Igf1*) mRNA in the cerebral cortex [5]. Because GH and IGF1 are the main hormones involved in lipid metabolism, we investigated the relationships between St3gal4 and lipid metabolism using different types of oils to determine the effects of oils on the emotional behaviors of KO mice [15]. Changes in emotional behaviors generated by consumption of a diet containing oil differed between St3gal4-KO mice and their wild-type (WT) littermates. When St3gal4-deficient mice were fed pellets containing saturated fatty acids, the mice showed decreased tone fear, reaching the level of that in WT mice. Thus, these data suggested that St3gal4 may be involved in both lipid metabolism and neuropsychiatric disorders. The metabolic rate of mice is roughly 7 times higher than that of humans [16], and glucose metabolism per gram of brain tissue in mice is 2.9 times higher than that in humans [17]. These suggest that the higher metabolic rate in mice increases the loading to neural functions. The use of depression and anxiety model mice offers an advantage in studying the effects of metabolism on psychiatric conditions. Hence, we decided to investigate the relationship between metabolic changes that occur in St3gal4-KO mice.

Metabolomics is the systematic characterization of the repertoire of small molecules (metabolites) in a tissue or organism and has been widely used recently owing to methodological advances [18–21]. Studies have shown that the metabolic profiles of biological fluids reflect differences in the endocrine state, age, and genetic makeup of the donors [22–25]. Urinary profiles have been well characterized for the diagnosis of disease [26,27]. Urine, an excreted body fluid, is readily available and easily collected using noninvasive methods. Urine is rich in chemical information and is a potent source of pheromones [28,29]. Among the several thousand components in urine [30], volatile urinary compounds represent nearly all chemical classes, including aldehydes, alcohols, ketone, and hydrocarbons [31]. Alterations in urine volatiles have been used to detect urinary tract infections as well as bladder, prostate, and other cancers [32].

Previously, we demonstrated that differential peripheral lipid metabolism was involved in determining emotional and cognitive behaviors. Although various small molecules are produced as a result of lipid metabolism, the relationships between their production and emotion have not been examined. In this study, we aimed to investigate metabolic changes related to metabolism in St3gal4-KO mice by directly evaluating volatile organic compounds (VOCs) as end-metabolites using solid-phase micro-extraction (SPME) to extract urinary VOCs [33] followed by gas chromatography-mass spectrometry (GC-MS) to screen for differential expression of urinary VOCs between St3gal4-KO and WT mice. Overall, our results provided insights into the roles of VOCs in neuropsychiatry and a deeper understanding of emotional responses.

## Materials and methods

### Ethics statement

All animals were treated in accordance with the Guidelines for Proper Conduct of Animal Experiments published by the Science Council of Japan (2006). The protocol was approved by the Committee on the Ethics of Animal Experiments of the Kyoto Sangyo University (approval no. 2017–08, 2018–07).

### Animals

*St3gal4*-deficient mice (C57BL/6-St3gal4<tm1.1Bsi>, RBRC02286) were generated previously [5]. Briefly, St3gal4-KO and WT mice carried a C57Bl/6J background and were obtained by genome editing with MS12 embryonic stem cells (derived from C57Bl/6J mice) via electroporation and backcrossing with C57Bl/6J mice. The experiments were conducted using male mice of the 13^th^ to 20^th^ generations. Mice were housed in a temperature-controlled room (22–24°C) with a 12-h light/dark cycle and were provided free access to food (MF; Oriental Yeast Co., Ltd, Tokyo, Japan.) and water. Four mice were maintained in open-top plastic cages (225 mm [width] × 338 mm [length] × 140 mm [height]). The cage tops were covered with stainless steel wire grid lids, and the cage floors were covered with paper-chips (Eco-chip; CLEA Japan, Inc., Tokyo, Japan), which were changed twice weekly. At the completion of the study mice were euthanized with isoflurane (Pfizer, NY, NY, USA).

### Chemicals

The following standard chemicals were used in this study: methylamine, *N*, *N*,-dimethyl (trimethylamine; 25% pure in ethanol; cat. no. T2892; Tokyo Chemical Inc., Tokyo, Japan); 3-penten, 2-one (70% pure; cat. no. 145017; Sigma, St. Louis, MO, USA); pentanoic acid, 4-methyl, ethyl ester (> 97% pure; cat. no. 58730; Sigma); styrene (99% pure; cat. no. S0095; Tokyo Chemical Inc.); 3-hepten-2-one (95% pure; cat. no. H0838; Tokyo Chemical Inc.); benzaldehyde (99.9% pure standard for quantitative nuclear magnetic resonance; cat. no. B1334; Sigma); 2,2,4-trimethyl-1,3-pentanediol 1-monoisobutyrate (texanol; cat. no. 40366; Alfa Aesar, Lancashire, UK); *n*-alkane mix solution (C9-C40: 50 μg/mL; C10, 20, 30, and 40: 100 μg/mL; cat. no. 102158321; GL Sciences Inc., Tokyo, Japan). β-Farnesene was kindly provided by Nippon Terpene Chemicals Inc. (Kobe, Japan).

### Collection of urine

Urine was collected from aged St3gal4-KO and WT littermate male mice at 20–35 weeks of age. Urine was collected from naïve male mice at 10–15 weeks of age before and after encounters with females. Collected urine was flash-frozen with liquid N_2_ and stored at -80°C until use. Urine collected daily from one mouse was combined in one tube, 200 μL aliquots were separated into 2-mL glass vials for analysis. Urinary creatinine concentrations were determined using a LabAssay^TM^ Creatinine colorimetry kit (Wako Pure Chemical Industries, Ltd., Osaka, Japan), which was based on the Jaffé method [34].

### Extraction of urinary volatile compounds by solid-phase microextraction

Urinary volatile compounds were extracted using headspace (HS)-SPME fibers. We used 2 cm 50/30 μM divinylbenzene/carboxen/polydimethylsiloxane (DVB/CAR/PDMS; Supelco, Bellefonte, PA, USA) as SPME fibers to cover a wide range of molecular weights. The fibers were preconditioned at 250°C for 30 min and inserted into the headspace of the vial after the vial was equilibrated for 1 min at 45°C. The volatile compounds in the headspace were extracted using SPME fibers at 45°C for 60 min as previously described [35,36].

### GC-MS

VOCs in middle-aged mice at 20–35 weeks were measured twice (experiment 1 and experiment 2) with a triple quadrupole GC-MS system (TQ-8040; Shimadzu, Kyoto, Japan). The SPME fiber with absorbed volatile compounds was inserted into the GC injection port and desorbed for 3 min at 240°C. The injection was pulsed splitless for 3 min. To separate the desorbed volatiles, InertCap PureWAX with ProGuard and T.L. column (60 m + 10 m pro-guard line and 2 m transfer line, 0.25 mm internal diameter, 0.5 μm film thick; GL science, Tokyo, Japan) was used as previously described by Fujita et al. [36]. The oven temperature was programmed as follows: held at 40°C for 10 min, ramped to 240°C at 5°C/min, and held for 10 min. The injector temperature was set at 240°C. Helium was used as carrier gas at a constant column flow rate of 2 mL/min for 0–3 min and 1 mL/min for 3–60 min. The operating parameters for MS were as follows: ion source temperature, 200°C; ionizing energy, 70 eV; scanning frequency, 0.3 s/spectrum from 35 to 300 *m/z* for experiment 1 and 0.2 s/spectrum for experiment 2. For urinary VOC analysis in mouse at 10 to 15 weeks of age, the procedure was the same as in experiment 2 except that the constant linear velocity of helium was changed to 20 cm/s by QP-2010 Ultra (Shimadzu). VOC identification was carried out based on comparisons of retention times with those of standards and with probability matching against a mass spectral library (NIST/EPA/NIH mass spectral library, NIST14; Mass Spectral Library of Drugs, Poisons, Pesticides, Pollutants, and Their Metabolites, Wiley10). The retention index (RI) of VOCs were calculated from retention time of the *n*-alkane standard and searched the data in NIST Chemistry WebBook [37]. The qualities of commercial texanol were analyzed using 1H NMR spectra of compounds recorded on a JEOL JNM-ECS 400 spectrometer with TMS as the internal standard [38] as texanol included two compounds, texanol and texanol isomer.

### Data processing and statistical analyses

The chromatographic peak areas were integrated using GC-MS Solution ver.4.45 software (Shimadzu). Detection and integration of the generated ion peaks from electron ionization was performed using XCMS software package version 1.3.2 (http://masspec.scripps.edu) [39,40] in R version 3.2.3 (http://cran.r-project.org/). XCMS software was used to process peak-matching, non-linear retention time alignment, and quantitation of mass spectral ion intensities. The total ion current (TIC) for each peak was manually inspected to validate the detected peak. Refinement of VOCs was carried out by manual deconvolution based on the retention times and peak shapes of the various extracted ion peaks (*m/z*).

First, differential urinary VOCs between St3gal4-KO and WT mice at 20–35 weeks of age were screened. VOCs in mice were measured with GC-MS three times. Triplicate urinary VOCs in St3gal4-KO (n = 3 in experiments 1; n = 3 in experiment 2) and WT (n = 3 in experiment 1; n = 3 in experiment 2) mice were examined by XCMS to detect differential ion peaks (Table 1). Finally, the area of the ion peaks was quantified using GC-MS Solution software. To compile each dataset (experiment 1 and experiment 2), we used the average of three absolute ion peak areas to calculate the relative intensity of the average area of KO mice relative to that of the WT littermates (n = 6 pairs). The absolute values of ion peak area from urinary VOCs were compared between young KO and WT mice (male, 10–16 weeks old). Differential levels of VOCs were analyzed using Excel and Prism 6 by Mann Whitney *U*-tests and Kruskal-Wallis tests.

**Table 1:** Identification of urinary volatile organic compounds (VOCs) showing different quantities between St3gal4-KO mice and WT littermate mice by XCMS.

No.	Mainly observed ion (*m/z*)	Quantified ion (*m/z*)^a)^	RI^b)^	Compound	SI^c)^	Chemical class	CAS No.	Chemical formula	Relative values of KO mice to WT (fold)	S.E.M	*p* value^d)^
**1**	42, 58, 59	58	<900	Methylamine, *N*,*N*-dimethyl^†^	97	Amine	75-50-3	C_3_H_9_N	0.467	0.117	**0.0022
**2**	57, 97, 126	57	955	RI955^e)^	87				1.227	0.156	0.3636
**3**	55, 85, 111	55	1101	RI1101^f)^	87				2.395	0.492	*0.0476
**4**	41, 43, 69	69	1128	3-Penten, 2-one^†^	92	Ketone	625-33-2	C_5_H_8_O	0.761	0.120	*0.0476
**5**	88, 99, 101	88	1174	Pentanoic acid, 4-methyl-, ethyl ester^†^	90	Fatty acid ester	25415-67-2	C_8_H_16_O_2_	7.083	3.083	0.2424
**6**	43, 57, 81	57	1198	3-Heptanone, 6-methyl	95	Ketone	624-42-0	C_8_H_16_O	12.900	6.484	*0.0476
**7**	41, 43, 94	43	1227	RI1227^g)^	90				1.820	0.519	*0.0476
**8**	41, 43, 94	43	1237	RI1237^h)^	90				2.559	0.546	**0.0022
**9**	78, 103, 104	104	1251	Styrene^†^	96	Benzene	100-42-5	C_8_H_8_	2.027	0.733	0.3636
**10**	41, 57, 69	57	1291	RI1291^i)^	90				4.585	1.454	**0.0022
**11**	43, 55, 97	55	1294	3-Hepten-2-one^†^	90	Ketone	1119-44-4	C_7_H_12_O	n.d.	n.d.	n.d.^j)^
**12**	77, 105, 106	106	1537	Benzaldehyde^†^	97	Aldehyde	100-52-7	C_7_H_6_O	2.770	0.943	**0.0022
**13**	41, 69, 93	69	1665	beta-Famesene^†^	95	Hydrocarbon	18794-84-8	C_15_H_24_	4.793	1.287	*0.0476
**14**	41, 93, 107	93	1749	alpha-Farnesene	97	Hydrocarbon	502-61-4	C_15_H_24_	5.918	1.939	*0.0476
**15**	56, 71, 89	71	1866	2,2,4-Trimethyl-1,3-pentanediol 1-monoisobutyrate (texanol)^†^	97	Hydrocarbon	25265-77-4	C_12_H_24_O_3_	1.288	0.147	0.3636
**16**	43, 71, 83	71	1886	2,2,4-Trimethyl-1,3-pentanediol 3-monoisobutyrate (texanol isomer)^#^		Hydrocarbon	77-68-9	C_12_H_24_O_3_	1.322	0.131	*0.0476
**17**	67, 69, 109	67	1939	5,9-Undecadien-2-ol, 6,10-dimethyl	91	l)	53837-34-6	C_13_H_24_O	14.401	8.269	*0.0368
**18**	43, 83, 97	43	2297	RI2297^k)^	95				2.667	1.046	0.2424

^a)^ The area of an ion peak was used for quantification of urinary VOCs.

^b)^ Retention indices of VOCs using InertCap PureWAX.

^c)^ Similarity indices (SI) show the similarities of mass spectra from the NIST 14 standard reference database, and VOCs with SIs of more than 85% are listed.

^d)^ Mann Whitney *U*-test (two-tailed).

^e)^ The NIST library identified 4-octen-3-one, 6-ethyl-7-hydroxy (ketone) as a TIC, and the maximum SI with the MS spectrum was 87%. However, RI information was not obtained from the NIST chemistry book.

^f)^ The NIST library identified 2-hexenal, 2-ethyl- (aldehyde) as a TIC, and the maximum SI with the MS spectrum was 87%. However, the retention time was not the same as that of the commercial standard.

^g)^ The NIST library identified 5-hexen-2-one, 5-methyl-/3-hexen-2-one, 5-methyl- (ketone) as a TIC, and the maximum SI with the MS spectrum was 90%. However, RI information was not obtained from the NIST chemistry book.

^h)^ The NIST library identified 5-oxohexanenitrile (ketone) as a TIC, and the maximum SI with the MS spectrum was 90%. However, the retention time was not the same as that of the commercial standard. The second hit from the NIST library was 5-hepten-2-one, and the SI with the MS spectrum was 89%. However, RI information was not obtained from the NIST chemistry book.

^i)^ The NIST library identified 3-heptanone, 5-methylene (ketone) as a TIC, and the maximum SI with the MS spectrum was 90%. However, RI information was not obtained from the NIST chemistry book.

^j)^ n.d., not determined. Since no ion peaks were detected in the urine of St3gal4-KO mice and WT littermates in experiment 2, the fold-change, SEM, and *p* value were not calculated.

^k)^ The NIST library identified eicosyl acetate (hydrocarbon) as a TIC, and the maximum SI with the MS spectrum was 95%. However, RI information was not obtained from the NIST chemistry book.

^l)^ The chemical class is shown as having a hydroxy group with only carbon-to-carbon double bonds as unsaturation, in Pubchem [PubChem Identifier: CID 5370125].

^†^VOCs were confirmed by identification using commercial standard references.

^#^ The NIST library identified 2,2,4-trimethyl-1,3-pentanediol diisobutyrate (TXIB) with 94% SI as a TIC. However, the MS spectrum of compound (16) did not include *m/z* 159 and *m/z* 243, which were detected in commercial TXIB by GC-MS. On the other hand, the commercial texanol standard included two peaks; one represented texanol, and the other showed the same retention time as compound (16) with a similar MS spectrum. Based on NMR analysis, the peak in the commercial texanol standard corresponding to compound (16) was determined to be texanol isomer, which we were unable to find in the NIST library.

Among multivariate statistical analyses, principal component analysis (PCA) score plots were obtained from relative values of 17 VOCs in six pairs of St3gal4-KO mice and WT littermates using Excel, KyPlot 5.0 (KyensLab Inc., Tokyo, Japan), and IBM SPSS Statistics Version 25 (Armonk, NY, USA). Hierarchical cluster analysis was conducted using absolute values of ion peak areas with IBM SPSS Statistics Version 25. A polynomial regression curve to evaluate whether VOCs were correlated with startle responses was established using a combination of Excel and IBM SPSS Statistics Version 25.

### Startle response

The acoustic startle response was tested in mice to observe the sensorimotor fright response using a startle reflex test unit for mice (O’Hara & Co., Ltd., Tokyo, Japan) [41]. A mouse was inserted to an acrylic chamber, the chamber was placed onto a sensor unit, and both the chamber and sensor unit were fixed using a clamp. The speakers conveyed continuous background noise of 70 dB (white-noise) for a 5-min acclimation period and throughout the test session. The mouse was then exposed to a 120-dB noise pulse for 20 ms per 500 ms 10 times. Vibrations caused by the whole-body startle response of the mouse were displayed numerically with the acceleration sensor. The numerical signal was amplified and converted into the voltage, which was displayed as the shape of waves. The peak-to-peak value of this wave was adopted as the strength of the startle response.

### Open field test

Open field tests for encounters with females were conducted on St3gal4-KO and WT male mice at 10–16 weeks of age. Each of the animals was acclimatized to being alone in a separate cage in a waiting room for at least 30 min before the behavioral sessions. Behavioral tests were performed between 0800 and 1300 by an experimenter blinded to the mouse genotype. The open field test was performed by placing the mice in a square-shaped arena (490 mm wide × 490 mm long × 195 mm high) that had a ceiling height 125 cm above the field floor. The light intensity of the fluorescent lamp that lit the open field and the acclimatization waiting room with the separate cage was set to 70 lux.

Female mice (ddY, 8–12 weeks old) underwent vaginal smear tests just before the open field tests to determine estrous cycle stages [42]. The smears were stained with Giemsa solution (Wako Pure Chemical Industries, Ltd.) and examined by Eclipse 80i bright-field microscopy (Nikon, Tokyo, Japan).

A male mouse was first placed in the field, and 1 min later, a female mouse in the proestrus period was placed in the field for 30 min. Both mice were then returned to their home cages. Data were collected with a camera placed 470 mm above the field floor using TimeOFCR4 (O’Hara & Co., Ltd.). After recording, the numbers of male accesses per 1 s from male nose to female nose, from male nose to female circumanal region, and from male hand to female body were counted for the first 10 min.

## Results

### Specific differences in urinary VOCs in St3gal4-KO mice

**Fig 1:**
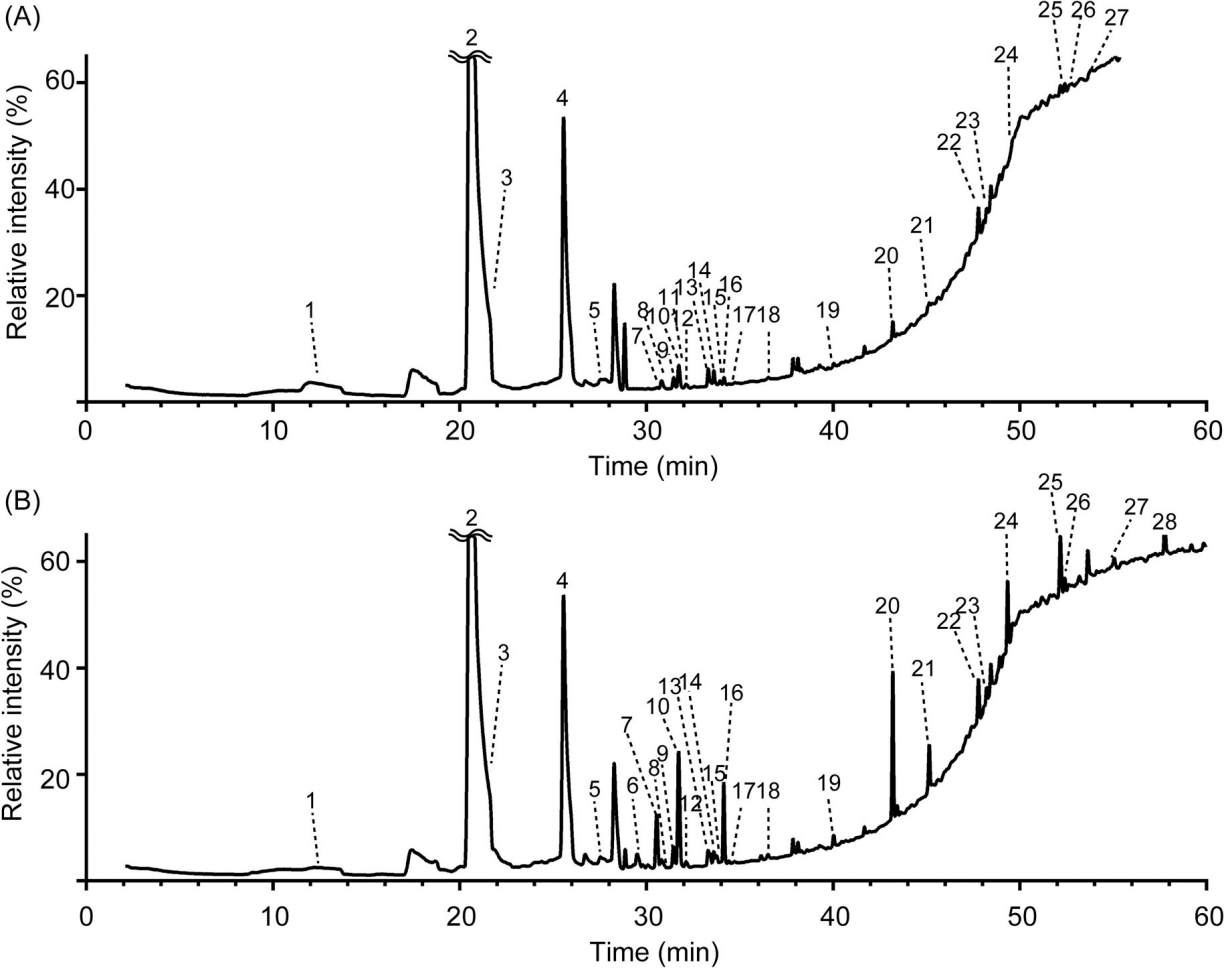
Typical GC-MS total ion (TIC) chromatograms of urinary volatile organic compounds (VOCs) in St3gal4 WT and KO mice. TIC chromatograms were obtained from analysis of the samples by HS-SPME and GC-MS, which were performed as described in Materials and Methods. The TIC chromatograms are shown as relative intensity when the absolute intensity of peak no. 2 is 100%. Numbers indicate the following metabolites that showed similarity indexes of above 85%: 1) methylamine, *N*,*N*-dimethyl-; 2) 4-octen-3-one, 6-ethyl-7-hydroxy-; 3) 2-pentanone; 4) 2-hexenal, 2-ethyl-; 5) ethanone, 1-cyclopropyl-; 6) 2-heptanone; 7) 3-heptanone, 6-methyl-; 8) 2-penten-1-ol, acetate, (Z)-; 9) 5-hexen-2-one, 5-methyl-; 10) 5-oxohexanenitrile; 11) 5-hepten-2-one; 12) 1,3,5,7-cyclooctatetraene; 13) 6-hepten-3-one, 4-methyl-; 14) 3-hepten-2-one; 15) pentane, 2-nitro-; 16) 3-heptanone, 5-methylene-; 17) 5-hepten-2-one, 6-methyl-; 18) dodecane, 2,6,11-trimethyl-; 19) benzaldehyde; 20) β-farnesene; 21) α-farnesene; 22) 2,2,4-trimethyl-1,3-pentanediol 1-monoisobutyrate (texanol); 23) 2,2,4-trimethyl-1,3-pentanediol diisobutyrate (TXIB); 24) 5,9-undecadien-2-ol, 6,10-dimethyl-; 25) p-cresol; 26) 2,5-cyclohexadien-1-one, 2,6-bis(1,1-dimethylethyl)-4-hydroxy-4-methyl-; 27) methyl octadecyl ether; 28) 1-docosanol, acetate (eicosyl acetate).

Urine samples from middle-aged mice at 20–35 weeks old (n = 3, KO, n = 3, WT in experiment 1; n = 3, KO, n = 3, WT in experiment 2) were analyzed using headspace-SPME GC-MS. The representative TIC peaks and list of VOCs estimated by a similarity index above 85% from experiment 1 and experiment 2 are shown in Figs 1 and S1. Forty-two VOCs were detected in experiment 1, and 75 VOCs were detected in experiment 2 (S1 and S2 Tables). These data indicated that the urine of C57Bl/6J mice contained at least 97 VOCs.

To screen differential VOCs between St3gal4-KO and WT mice, GC-MS data sets were analyzed by XCMS. By alignment, we obtained information for each ion peak as to retention time, *m/z* value, *p* value, and fold change (S3 and S4 Tables). XCMS detected 558 and 729 ion peaks in experiments 1 and 2, respectively. Ion peaks with *p* values of less than 0.05 were defined as differentially extracted ion peaks (S3 and S4 Tables). From each extracted-ion chromatogram, 93 and 244 differentially extracted ion peaks in experiments 1 and 2, respectively, were selected for further investigation. By deconvolution analysis, 18 VOCs were isolated using the retention time of each peak and a similarity cutoff of 85% (Table 1). Nine (labeled with a dagger symbol in Table 1) out of the 18 VOCs—methylamine, *N*,*N*-dimethyl (compound 1, as shown in Table 1); 3-penten, 2-one (compound 4); pentanoic acid, 4-methyl-, ethyl ester (compound 5); styrene (compound 9); 3-hepten-2-one (compound 11); benzaldehyde (compound 12); beta-farnesene (compound 13); 2,2,4-trimethyl, 1,3-pentandiol, 1-monoisobutylate (texanol) (compound 15); and 2,2,4-trimethyl,1,3-pentandiol, 3-monoisobutylate (texanol isomer) (compound 16)—were confirmed by matching the mass spectra of commercially available reagents. Three out of 18 VOCs—3-heptanone, 6-methyl (compound 6); alpha-farnesene (compound 14); and 5,9-undecadien-2-ol, 6,10-dimethyl (compound 17)—had high similarity to the database and were identified by matching RIs. RI955 (compound 2), RI1101 (compound 3), RI1227 (compound 7), RI1237 (compound 8), and RI1291 (compound 10) are shown as calculated RIs because their RI information was not obtained from the NIST chemistry book [37]. Next, to evaluate if differential areas of ion peaks belonging to the 18 VOCs were correct, the base peaks (Table 1) of candidate VOCs were selected (excluding 3-hepten-2-one, which was undetectable in experiment 2), and the areas of the ion peaks in the VOCs were quantified using GC-MS Solution; significance was determined by Mann-Whitney *U*-tests. Methylamine, *N*,*N*-dimethyl (compound 1) and 3-penten, 2-one (compound 4) were downregulated, whereas RI1101 (compound 3); 3-heptanone, 6-methyl (compound 6); RI1227 (compound 7); RI1237 (compound 8); RI1291 (compound 10); benzaldehyde (compound 12); β-farnesene (compound 13); α-farnesene (compound 14); texanol isomer (compound 16); and 5,9-undecadien-2-ol, 6,10-dimethyl (compound 17) were upregulated in St3gal4-KO male mice, which represented the anxiety and depression model.

### Metabolomic characterization of urinary VOCs by GC-MS

**Fig 2:**
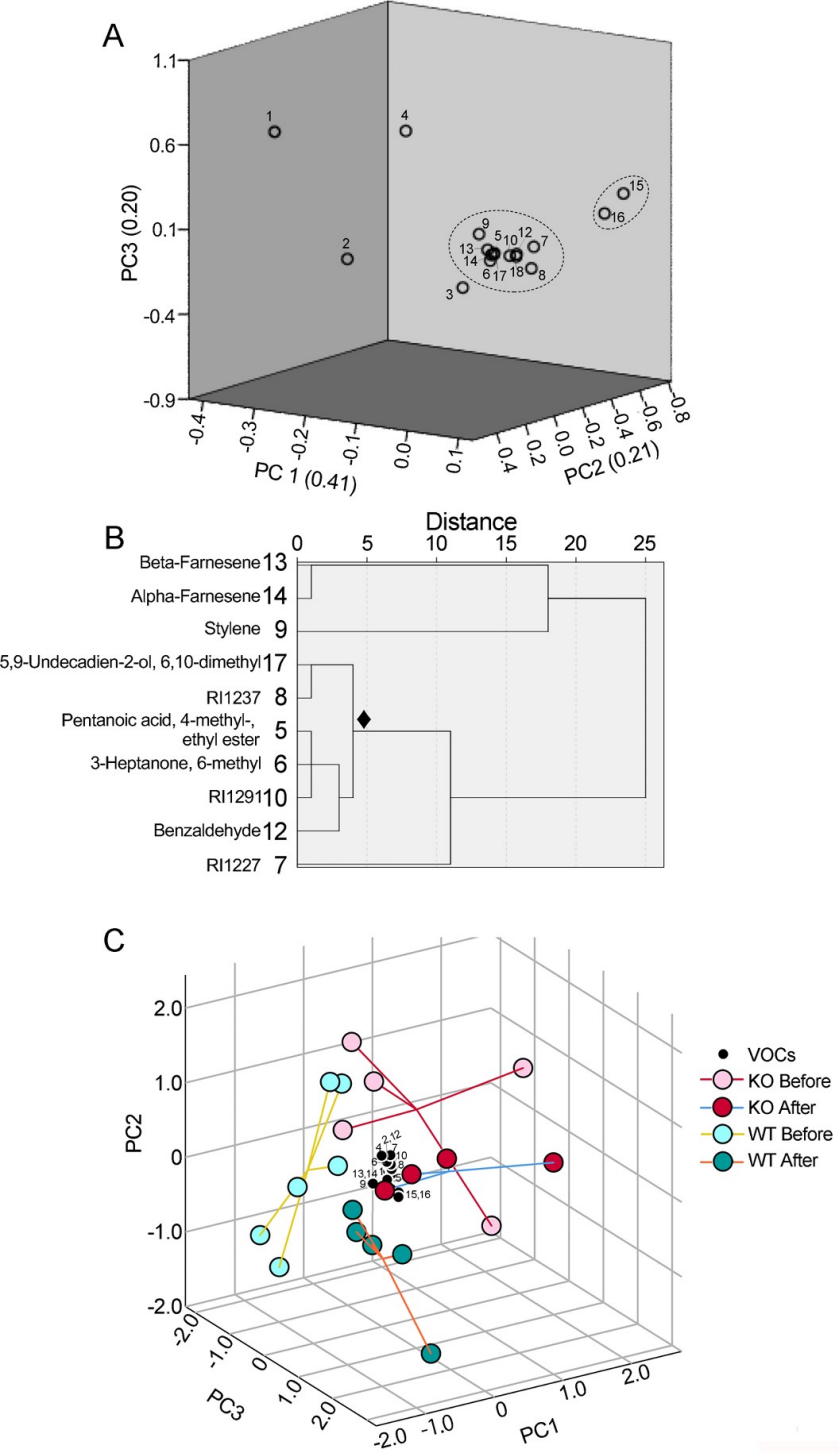
Extracts of a group by PCA score plot of 17 VOCs, classification of each VOC by hierarchical cluster analysis, and separation of four different groups of young mice. PCA score plots were derived from the values of 17 VOCs in middle-aged St3gal4-KO mice relative to those in WT littermates for six paired mice (A). Contribution ratios were 0.415 for PC1, 0.214 for PC2, and 0.201 for PC3 (total of 0.829). The number of contours in Fig 2 is the same as the number in the first column in Table 1. One group included 11 VOCs: pentanoic acid, 4-methyl-, ethyl ester (5); 3-heptanone, 6-methyl (6); RI1227 (7); RI1237 (8); styrene (9); RI1291 (10); benzaldehyde (12); β-farnesene (13); α-farnesene (14); 5,9-undecadien-2-ol, 6,10-dimethyl (17); and RI2297 (18) (Table 1). Another group included two VOCs: 2,2,4-trimethyl-1,3-pentanediol 1-monoisobutyrate (texanol) (15) and 2,2,4-trimethyl-1,3-pentanediol 3-monoisobutyrate (texanol isomer) (16). A hierarchical clustering analysis dendrogram featuring 10 VOCs [excluding RI2297 (18)] in Fig 2A was created from PC1 to PC6 (cumulative contribution ratio = 0.986), obtained using absolute area values of ion peaks in St3gal4-KO (n = 9) and WT (n = 11) mice by PCA (B). PCA score plots were derived from the absolute values of 14 VOCs from young mice urine samples presented in Table 2, in which RI1101 or 5,9-undecadien-2-ol, 6,10-dimethyl was excluded due to the presence of mice showing below detection limit of the GC-MS (C). PC1 to PC3 scores from mice as well as PC1 to PC3 standardized scoring coefficients (black circles) in the principal component score coefficient matrix for each VOC were represented as the X-, Y-, and Z-axis, respectively. Circles with a perpendicular line to the center of gravity are pink and red in color for *St3gal4*-KO mice before and after social interactions; while light green and green represent WT mice before and after social interactions, respectively.

To evaluate the correlations between VOCs using all ion peaks of St3gal4-KO and WT mice, PCA was performed using relative values of base-peaks in 17 VOCs (excluding 3-heptene-2-one from the set of 18 VOCs) in St3gal4-KO and WT mice (Table 1 and Fig 2A). The cumulative contribution proportion should be more than 0.8, and the value in our study was 0.829 based on the sum of principal components 1 (PC1) to 3 (PC3). Therefore, a PCA score plot including the X-, Y-, and Z-axes was displayed, resulting in the clustering of one major group composed of 11 VOCs—pentanoic acid, 4-methyl-, ethyl ester (5); 3-heptanone, 6-methyl (6); RI1227 (7); RI1237 (8); styrene (9); RI1291 (10); benzaldehyde (12); β-farnesene (13); α-farnesene (14); 5,9-undecadien-2-ol, 6,10-dimethyl (17); and RI2297 (18)—as well as a group of two VOCs [texanol (15) and texanol isomer (16)] and other VOCs [methylamine, *N*,*N*-dimethyl (1); RI955 (2); RI1101 (3); and 3-penten, 2-one (4)] (Fig 2A).

**Table 2:** Absolute values of ion peaks in male urinary VOCs before and after encountering female mice at the proestrus stage.

		Absolute area of ion peak^a)^ in urine before access						Absolute area of ion peak^a)^ in urine after access					
		St3gal4-KO (n = 5)		Wild-type (n = 6)			M-W *U*-test	St3gal4-KO (n = 4)		Wild-type (n = 5)			M-W *U*-test
No.	VOCs	Average (x10^3^)	S.E.M. (x10^3^)	Average (x10^3^)	S.E.M. (x10^3^)	Fold	*p* value^b)^ 2T(1T)	Average (x10^3^)	S.E.M. (x10^3^)	Average (x10^3^)	S.E.M. (x10^3^)	Fold	*p* value^b)^ 2T(1T)
1	Methylamine, *N*,*N*-dimethyl	19060	5776.0	20550	2276.0	0.93	> 0.9999 (0.5000)	32600	10080	32960	7690.0	0.99	> 0.9999 (0.5000)
2	RI955	26360	2316.0	15090	2836.0	1.75	*0.0303 (*0.0152)	25460	1827.0	14610	3379.0	1.74	0.1111 (0.0556)
3	RI1101	161.04	29.889	34.594	13.234	4.65	**0.0043	155.66	33.423	88.752	32.594	1.75^#^	0.1111 (0.0556)
4	3-Penten-2-one	583.01	102.40	542.67	105.88	1.07	0.9307	494.17	59.804	455.98	80.611	1.08	0.9048
5	Pentanoic acid, 4-methyl-, ethyl ester	26.127	12.034	4.0630	1.2700	6.43	0.0519 (*0.0260)	24.465	11.001	1.5680	0.53940	15.60^$^	*0.0159 (**0.0079)
6	3-Heptanone, 6-methyl	1153.0	467.60	187.99	41.286	6.13	*0.0173 (**0.0087)	471.98	216.40	84.043	11.859	5.62	*0.0159 (**0.0079)
7	RI1227	1155.0	245.86	559.99	209.231	2.06	0.1255 (0.0628)	1104.0	207.01	416.07	90.000	2.65	*0.0317 (*0.0159)
8	RI1237	4349.0	1669.0	1418.0	382.82	3.07	0.0823 (*0.0411)	4528.0	2230.0	1281.0	146.55	3.53	*0.0159 (**0.0079)
9	Styrene	10.500	2.0460	10.527	2.2300	1.00	0.9307 (0.4654)	5.0530	0.57120	6.1610	0.51750	0.82	0.1905 (0.0952)
10	RI1291	1399.0	457.55	338.04	86.564	4.14	**0.0043 (**0.0022)	950.15	340.63	257.51	66.054	3.69	*0.0317 (*0.0159)
12	Benzaldehyde	144.26	15.758	68.487	11.331	2.11	**0.0087 (**0.0043)	116.37	25.889	74.633	6.2780	1.56	0.0635 (*0.0317)
13	beta-Farnesene	17.455	5.1320	6.0270	1.2530	2.90	0.0823 (*0.0411)	13.416	6.4520	14.757	5.4650	0.91^#^	> 0.9999 (0.5000)
14	alpha-Farnesene	4.8990	1.5630	1.4140	0.41820	3.46	0.0519 (*0.0260)	3.1200	1.4120	3.8280	1.3270	0.82^#^	0.7302 (0.3651)
15	2,2,4-Trimethyl-1,3-pentanediol 1-monoisobutyrate (texanol)	116.12	23.037	82.821	8.8100	1.40	0.3290 (0.1645)	229.96	15.190	217.195	26.585	1.06	0.7302 (0.3651)
16	2,2,4-Trimethyl-1,3-pentanediol 3-monoisobutyrate (texanol isomer)	121.69	20.453	85.571	7.8490	1.42	0.1775 (0.0887)	170.70	10.273	147.58	17.667	1.16	0.5556 (0.2778)
17	5,9-Undecadien-2-ol, 6,10-dimethyl	33.299	19.004	4.2580	1.1910	7.82	0.1255 (0.0628)	24.535	20.957	1.5010	0.77390	16.35^$^	0.1032 (0.0556)
		Average (x1)	S.E.M. (x1)	Average (x1)	S.E.M. (x1)	Fold	*p* value^b)^ 2T(1T)	Average (x1)	S.E.M. (x1)	Average (x1)	S.E.M. (x1)	Fold	*p* value^b)^ 2T(1T)
	Creatinine (mg/dL^c)^	53.47	7.233	35.61	3.433	1.50	0.1126 (0.0563)	71.10	3.116	56.41	3.989	1.26	0.0556 (*0.0317)

VOCs in male urine samples collected before and after encounters with females at the proestrus/estrus stages were compared between St3gal4-KO and wild-type mice at 10–16 weeks of age. The order number (NO.) is the same as that in Table 1.

As orders of areas differed among VOCs, the number of significant digits was unified to five columns.

^#^ Slight difference in quantity in male urine after encounters with females at the proestrus stage.

^$^ Increase in VOC quantity after encounters with females.

^a) ^The ion peak (*m/z*) extracted in Table 1 was used to calculate the absolute area of the ion peak.

^b) ^Mann Whitney *U*-test: 2T, two-tailed; 1T, one-tailed. Two-tailed (one-tailed) *p* values were used to evaluate significant correlations.

^c)^ Urinary creatinine concentrations were determined by LabAssay^TM^ Creatinine colorimetry kits based on the Jaffé method [34].

To investigate which screened VOCs were related to emotional behaviors, we performed the startle test and social open field test and analyzed urine samples collected before and after these social behavior tests from St3gal4-KO (n = 9) and WT (n = 11) male mice at 10–16 weeks of age (Table 2). In the young male mice, RI2297 (18) was undetectable. The remaining VOCs were used for further analysis. To evaluate kidney glomerular filtration, we measured urinary creatinine, which exhibited minimal differences between St3gal4-KO and WT mice.

Hierarchical cluster analysis (Fig 2B) was performed to determine the relationships of the 10 VOCs (after excluding RI2297 from the set of 11 VOCs)—pentanoic acid, 4-methyl-, ethyl ester (5); 3-heptanone, 6-methyl (6); RI1227 (7); RI1237 (8); styrene (9); RI1291 (10); benzaldehyde (12); β-farnesene (13); α-farnesene (14); and 5,9-undecadien-2-ol, 6,10-dimethyl (17)—in the major group in Fig 2A. First, PCA scores using absolute values of 10 VOCs obtained from the young mice were determined. Then, a dendrogram of 10 VOCs was created using PC1–6 (0.986 cumulative contribution proportion). As a result, two categories were refined as a group: one including styrene (9), β-farnesene (13), and α-farnesene (14), and another group composed of six VOCs (hereafter referred to as ‘six VOCs’) including pentanoic acid, 4-methyl-, ethyl ester (5); 3-heptanone, 6-methyl (6); RI1237 (8); RI1291 (10); benzaldehyde (12); and 5,9-undecadien-2-ol, 6,10-dimethyl (17), with a subgroup of RI1227 (7) (Fig 2B). The shorter distances showed a high similarity in the differential patterns of VOCs in urine. Furthermore, PCA was performed for four different groups of young mice (Table 2): WT mice before encountering females; St3gal4-KO before encountering; WT after encountering; and St3gal4-KO mice after encountering (Fig 2C). The 14 VOCs from Table 2 (excluding RI1101 or 5,9-undecadien-2-ol, 6,10-dimethyl) were used for calculation since excluded two VOCs were below detection limit of the GC-MS in some mice. The percentage of variance explained by the three principal components were: 49% for PC1, 17% for PC2, and 16% for PC3 (total 82%). This suggests that the VOCs identified effectively created four groups based on genotype and social behavior. Further, the loadings of the 14 VOCs on the three PCs were concentrated near the origin in the PCA scores plot.

### Correlations between startle response and production of urinary VOCs

**Fig 3:**
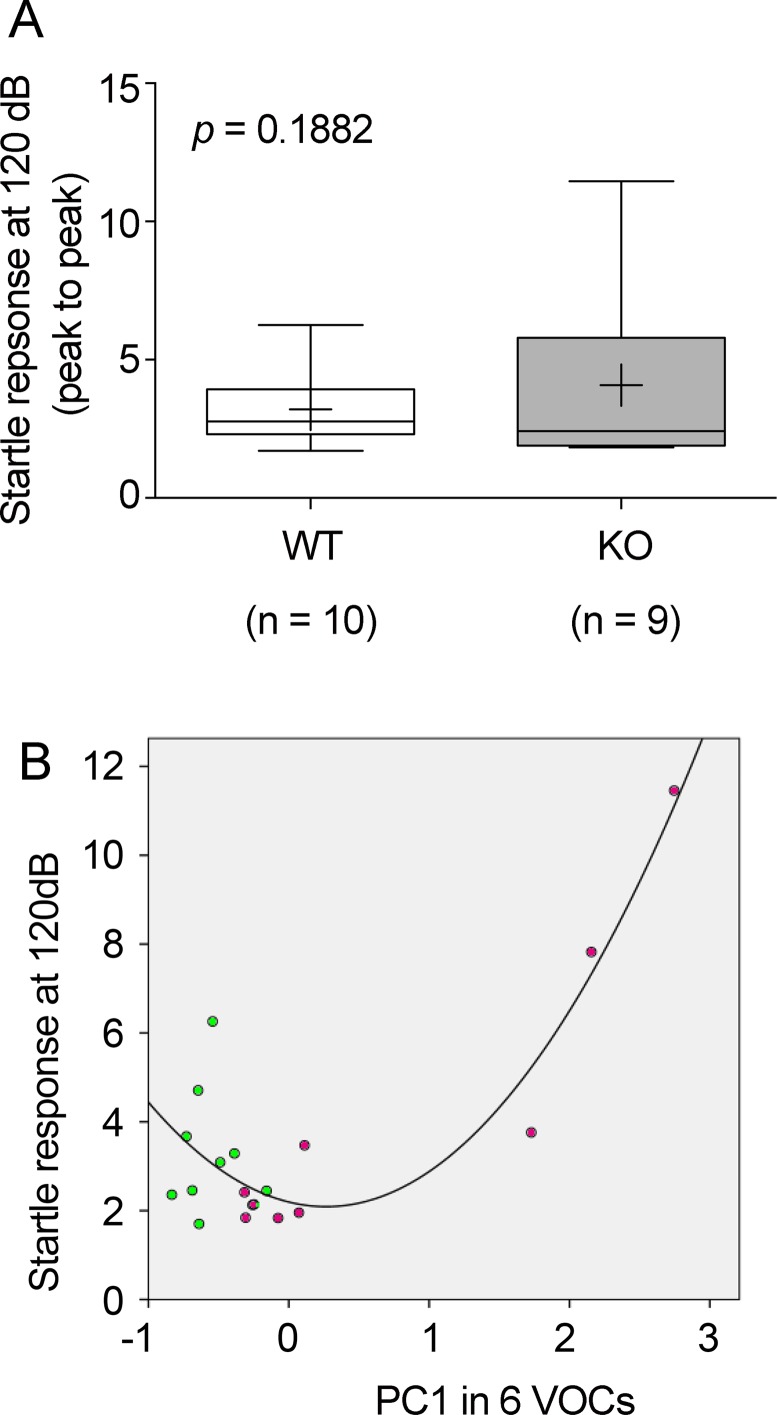
Correlation between startle response and PC1 from PCA score plot with the ‘six VOCs’. Startle responses at 120 dB for St3gal4-KO and wild-type (WT) mice (A). Mann Whitney *U*-test: *p* = 0.1882. Second-order polynomial regression curves between startle response and PC1 (0.898) of the ‘six VOCs’ (B): pentanoic acid, 4-methyl-, ethyl ester (5); 3-heptanone, 6-methyl (6); RI1291 (10); benzaldehyde (12); RI1237 (8); 5,9-undecadien-2-ol, 6,10-dimethyl (17). purple, St3gal4-KO mice; green, WT mice. *F* (2,16) = 29.336, ****p = 4.44*E* – 006, *r* = 0.886 (multiple correlation coefficient), *Y* = 2.1942 – 0.7805 * *x* + 1.4666 * *x*^2^.

Next, to analyze correlations between urinary VOC quantities and emotional response, we used a startle response test. First, we confirmed there were few differences in auditory activity or startle response between St3gal4-KO and WT mice (*p* = 0.1882 by Mann-Whitney *U*-tests in Fig 3A). Urine was collected after the startle response test. Second, to investigate the correlations between urinary VOC quantities and startle response, PCA scores using absolute values of the ‘six VOCs’ classified in Fig 2B were constructed. Because the percentage of variance contribution in the ‘six VOCs’ was captured 89.8% by PC1, which was very high, PC1 and startle response were distributed on the XY axis, resulting in a second-order polynomial regression curve with high fitting (*r* = 0.886; Fig 3B). Thirty-three percent of St3gal4-KO mice produced higher amounts of the ‘six VOCs’ in the urine and exhibited strong correlations between the production of the ‘six VOCs’ and startle response, whereas the remaining 67% of KO mice showed low amounts of the ‘six VOCs’. There was little correlation between production of the ‘six VOCs’ and the startle response in WT mice (Fig 3B).

### Correlation between access of male mice to female mice and production of urinary VOCs

**Fig 4:**
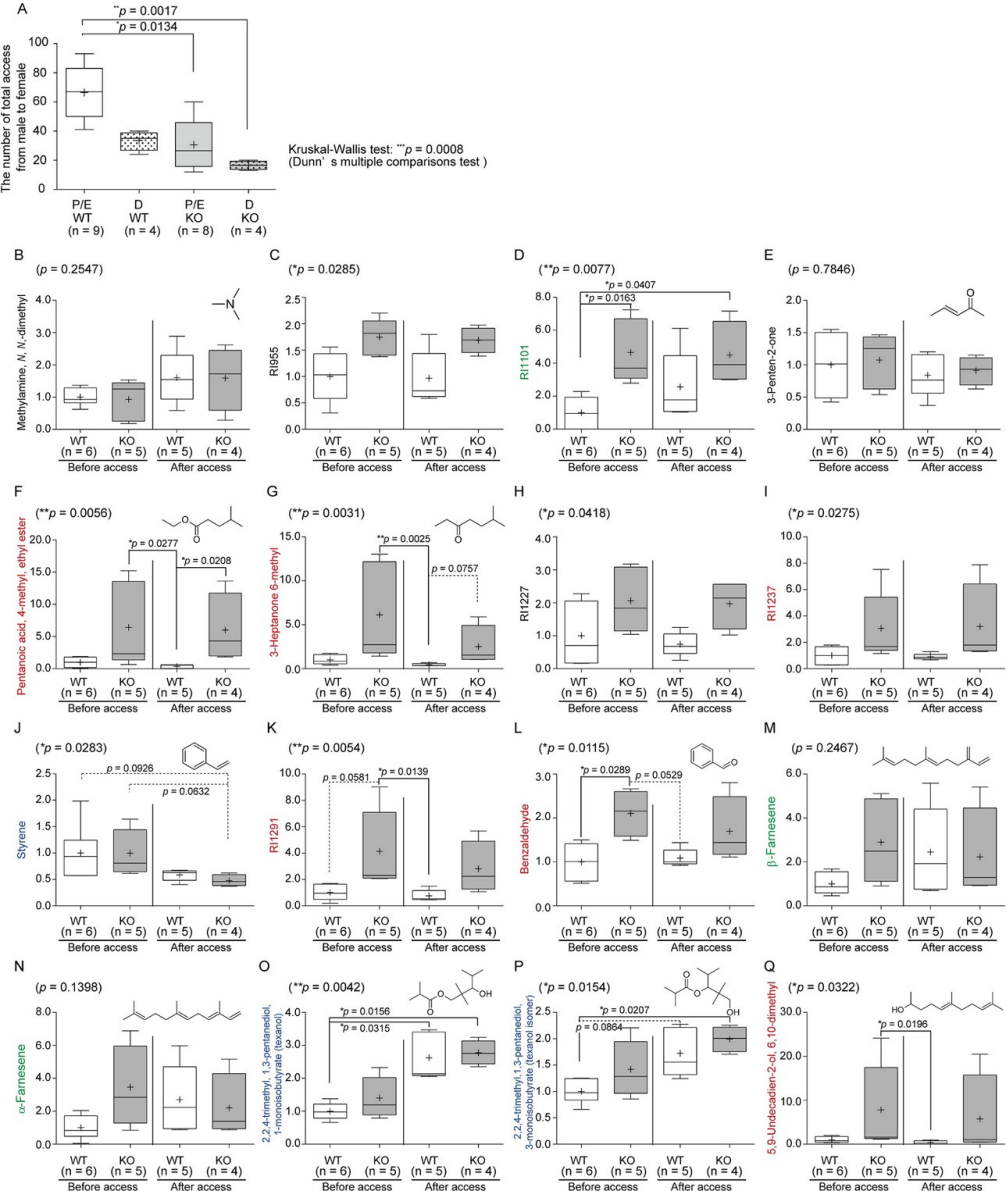
Comparisons of amounts of VOCs in urine collected from male mice before and after encounters with female mice at the proestrus and estrus stages in the open field test cage. Access of wild-type (WT) male mice to female mice at the P/E stage (A). The vertical line shows the number of male accesses at the P/E and diestrus stages per 1 s for 10 min. Different quantities of urinary VOCs were compared between St3gal4-KO and WT mice and between urine collected before and after encounters with female mice (B–Q). Urine was collected for 1 week before and after encounters with female mice. On the Y-axis, red indicates that St3gal4-KO mice exhibited higher amounts of VOCs in the urine than WT mice with low variance; green indicates that there were low amounts of VOCs in WT mice before encounters with female mice; and blue indicates that the concentrations of VOCs were changed in both St3gal4-KO and WT mice after encounters with female mice. Kruskal-Wallis test and Dunn’s multiple comparisons tests were used. WT, wild-type; KO, St3gal4-deficient; P/E, proestrus/estrus; D, diestrus stages.

To analyze another correlation between urinary VOC quantities and emotional responses, we performed social behavior tests (Fig 4A). The access of sexually naïve male mice to female mice at diestrus did not differ between WT and KO mice. The number of accesses of WT male mice to female mice at the proestrus/estrus (P/E) stage was increased 2.0-fold (means) compared with accesses to females at the diestrus stage, while accesses of St3gal4-KO male mice did not change regardless of female stage. This indicates that St3gal4-KO males failed to access females, even when female mice were in the P/E stage. Hence, we analyzed the effects of encounters with female mice at the P/E stage on male urinary VOC quantities to observe the emotional response.

**Fig 5:**
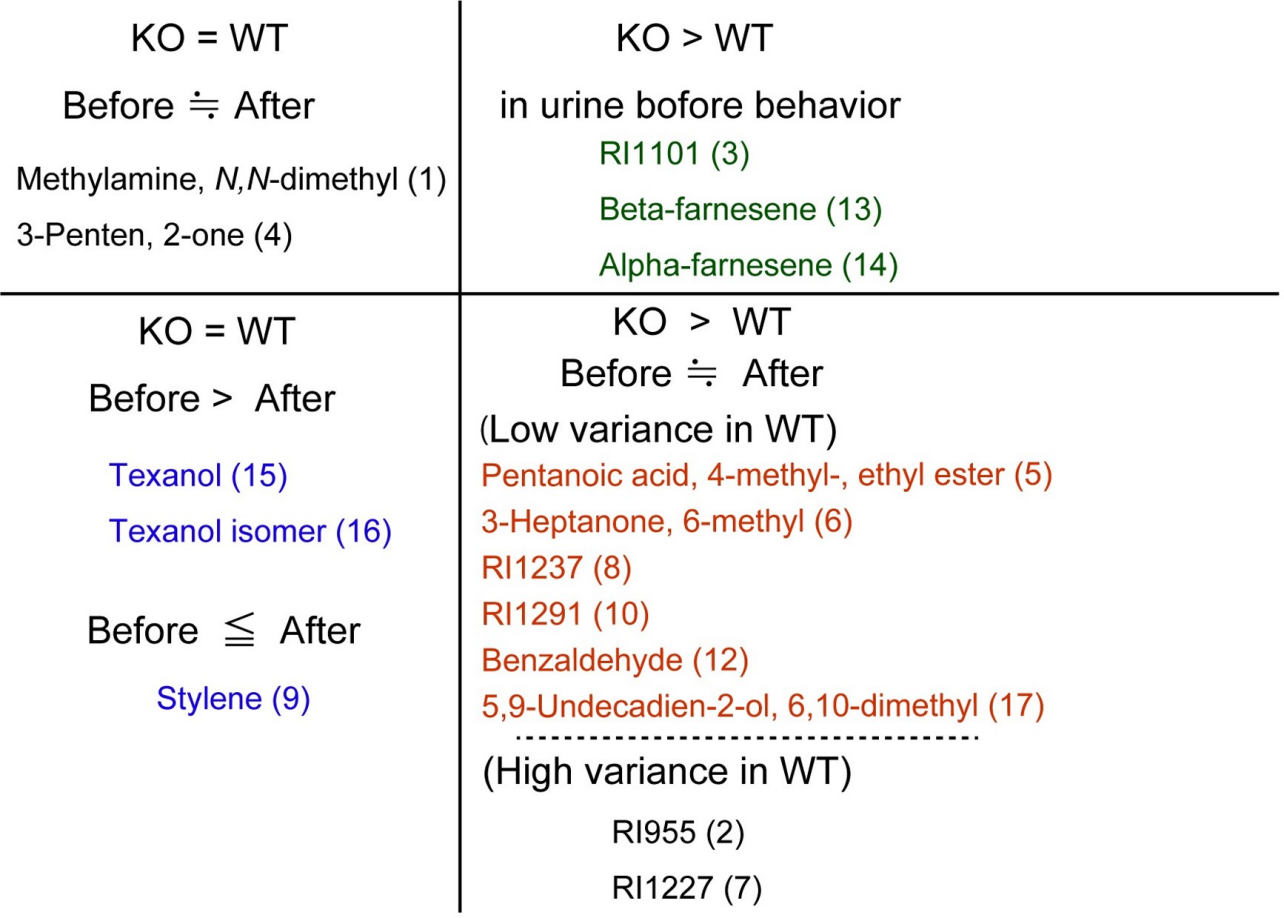
Summary of the differential quantities of VOCs. Numbers show those in Table 1. Names of VOCs written in red, green, and blue are identical to those written on the vertical lines in Fig 4.

Urine was collected from male mice before and after encounters with female mice at the P/E stage, and the analyzed VOCs are shown in Table 2. Following Kruskal-Wallis test, multiple comparison analysis was performed using Dunn’s test and by controlling the false discovery rate [43] (S5 Table). The trends by two multiple comparisons tests were similar. We found that there were three patterns of VOCs showing significant correlations with the number of male accesses. The first was RI1101 (3), β-farnesene (13), and α-farnesene (14), which were present at high levels in the urine of KO mice compared to those in WT mice before access (Mann–Whitney *U*-test, two-tailed *p* = 0.0043, one-tailed *p* = 0.0411 and 0.0260, respectively). After encountering female mice, these VOCs increased in WT male mice to the levels observed in KO mice (green in Figs 4D, 4M, 4N and 5 and Table 2), while the levels in KO mice after the encounters were the same as before the encounters. The second group was styrene (9), texanol (15), and texanol isomer (16), the levels of which changed after encounters with female mice in both St3gal4-KO and WT male mice (*p* = 0.0283, 0.0042, and 0.0154, respectively; blue in Figs 4J, 4O, 4P and 5 and Table 2). Styrene (9) decreased after encounters with female mice in the urine of both St3gal4-KO and wild-type male mice. Texanol (15) increased 2.0- and 2.6-fold after encounters with female mice in the urine of St3gal4-KO and WT mice, respectively, whereas texanol isomer (16) increased 1.4- and 1.7-fold, respectively.

Notably, the third group, termed the ‘six VOCs’, which were identified by hierarchical cluster analysis, were correlated with the startle response in the urine of St3gal4-KO mice and were higher than those in WT mice, regardless of encounters with female mice (red in Figs 4F, 4G, 4I, 4K, 4L, 4Q and 5 and Table 2). In other words, encounters with female mice did not elevate the amounts of the ‘six VOCs’. Furthermore, the absolute values of ion peaks for the ‘six VOCs’ from WT mice showed low variance (Fig 4F, 4G, 4I, 4K, 4L and 4Q).

## Discussion

### Identification of 18 VOCs

In the present study, we sought to determine whether St3gal4-KO mice showed variations in volatile urinary chemicals. These VOCs were extracted at 45°C for 60 min in the headspace and were concentrated on SPME fiber. Within these conditions we were unable to detect any non-volatile urinary compounds such as the major urinary proteins (MUPs) secrete by mice. These proteins harvest and sequester urinary nonpolar VOCs in hydrophobic pockets [44] and function as transporters and stabilizers of pheromones in rodents [45,46]. However, the ability to detect VOCs bound to MUPs may be limited in the current study and thus, we only effectively identified those that were unbound. Nevertheless, via GC-MS analysis, we detected 97 VOCs from middle-aged St3gal4-KO and WT mice. Among these VOCs, 18 VOCs were screened as significantly changed in St3gal4-KO mice by XCMS analysis.

PCA and hierarchical cluster analysis revealed three groups: group 1, termed the ‘six VOCs’; group 2, including β-farnesene (13) and α-farnesene (14); and group 3, including texanol, texanol isomer, additionally styrene. We investigated the relationship of these groups with emotional responses.

### Characterization of six VOCs

To examine the influence of the ‘six VOCs’ in the auditory thalamic sensory relay nuclei, where St3gal4 is specifically expressed [13], we investigated the startle response, an unconscious defensive response to threatening stimuli [41] and analyzed urinary VOCs collected after the startle experiment. These ‘six VOCs’ were elevated in St3gal4-deficient mice, and showed a correlation with the startle response in St3gal4-KO mice. The startle response is related to the auditory pathway, in which neural circuits and sound information is transmitted to the cochlear nucleus (CN). The auditory neurons in the CN ascend to the superior olivary complex (SO), and the neurons of the SO send axons to the inferior colliculus (IC) of the midbrain. The neurons of IC send axons to the medical geniculate body (MGN), and the axons of the MGN send axons to the auditory cortex [47]. The deficiency of *St3gal4*, which is specifically expressed in the MGN, appeared to influence the expression of the ‘six VOCs’. Further, the abundance of urinary VOCs showed variability in each St3gal4-KO mouse and exhibited large variance in Fig 4 in addition to Fig 3A. This high variability implies that St3gal4 deletion leads to dysregulation of the ‘six VOCs’ metabolism. Additionally, a portion of the St3gal4-KO mice that exhibited a startle response correlated with ‘six VOCs’ abundance, causing variability in Fig 3A. These suggested that the amounts of VOCs synchronously progress to out of regulation with behaviors in a part of St3gal4-KO mice.

Moreover, a previous study reported that within a bacterial metabolic network, functionality and constraints drive the tradeoff between robustness and fragility with high variability [48]. St3gal4 is a sialyltransferase and in humans SNPs or gene variants of ST3GAL4 affect lipid traits as well as the subsequent development of coronary artery disease [11,12]. Thus, further investigation is required to better understand how lipid metabolism alterations induced by St3gal4 deficiency affect the high variability of metabolic networks.

### Classification of VOCs by genotype and social behaviors in young mice

The number of accesses of male KO mice to female mice was unchanged by estrous stage, whereas the number of accesses of male WT mice to female mice in the P/E stage was increased 2.2-fold compared with that in the diestrus stage. We investigated the effects of social behavior in WT and KO mice on urinary VOCs. Encounters with female mice altered the amounts of urinary VOCs in naïve male mice. These VOC quantities effectively separated groups by genotype as well as by those from before and after social behavior by PCA. β-Farnesene and α-farnesene levels in group 2 were higher in KO mice than in WT mice before social interaction, whereas there was little difference after social interaction. These compounds are pheromones that are produced in males and have roles in attraction to females and puberty acceleration [49,50]. Our data showed that the amounts of urinary farnesenes were regulated in WT mice, but not KO mice, as a response to social behaviors. Encounters with female mice increased levels of group 3 compounds texanol and texanol isomer levels, regardless of genotype, while decreasing urinary styrene (Figs 4O, 4P and 5). It is surprising that these non-pheromonal VOCs were influenced by emotional behaviors. The ‘six VOCs’ were not correlated with the access of sexually naive male mice to female mice at the P/E stage. This suggests that urinary VOCs are differentially generated in response to emotional behaviors. Thus, our findings indicate that end metabolic products reflect emotional consequences.

It remains unclear how the levels of these VOCs are altered in response to emotional behavior. Further studies are also needed to determine how VOCs are produced and are involved in emotional behavior. VOCs including pheromones are likely to influence to emotion via the olfactory system, including the anterior olfactory nucleus, olfactory tubercle, piriform cortex, and cortical amygdala [51]. Table 3 lists the VOCs that were screened in St3gal4-KO mice, along with their chemical classes and whether they have been reported in mice and humans. These chemical classes include ketones, aldehydes, and hydrocarbons. Specifically, the lipid metabolism-related VOCs presented in Tables 1 and 2 included: pentanoic acid, 4-methyl, ethyl ester (5); 3-heptanone, 6-methyl (6); or β-farnesene (13) and α-farnesene (14), which were classified as ester, ketone, or hydrocarbon, respectively. Ketones are generated from the catabolism of medium chain fatty acids. Ketones in urine are likely partially derived from bacterial action in the gut. In fact, Holland et al found that ketones were produced in conventional urine yet were decreased in germ free mice [52]. Since urine was not collected from germ free mice in the current study, ketones may have been produced. Hydrocarbons produced via fatty acids are the products of lipid peroxidation. Alpha-farnesene and beta-farnesene are known to be produced from acetyl-CoA via the mevalonate pathway of cholesterol synthesis [53]. Additionally, 5,9-undecadien-2-ol, 6,10-dimethyl has been described in the Pubchem database as unsaturated with only carbon-carbon double bonds and a hydroxy group [PubChem Identifier: CID 5370125, 54]. It is also produced via the mevalonate pathway [55,56]. Therefore, 5,9-undecadien-2-ol, 6,10-dimethyl is included as lipid metabolism-related VOC in Tables 1 and 2. Since VOCs are the end products of endogenous and exogeneous pathways they often serve as biomarkers. In fact, multiple studies have identified different VOCs as markers of increased/dysregulated lipid metabolism [27,57,58]. Here it is inferred that ST3Gal IV influences VOC production by altering lipid metabolism.

**Table 3:** The roles of urinary VOCs in mice.

No.^a)^	Compound	Chemical class	Depression/anxiety model^b)^	Mouse reference	Human Metabolome Database [64]
1	Methylamine, *N*,*N*-dimethyl-	Amine	Decrease	Osada 2008 [65], Schaefer 2010 [25]	Blood, CSF, Feces, Saliva, Urine
4	3-Penten, 2-one	Ketone	Decrease	Kwak 2008 [66]	Feces Saliva
5	Pentanoic acid, 4-methyl-, ethyl ester	Fatty acid ester	Increase	N.D.	Feces
6	3-Heptanone, 6-methyl-	Ketone	Increase	Kwak 2008 [66], Schaefer 2010 [25]	N.D.
9	Styrene	Benzene	Increase	N.D.	Blood, Feces, Saliva
11	3-Hepten-2-one	Ketone	n.d.	Osada 2008 [65], Arnaiz 2014 [67]	Culture cell
12	Benzaldehyde	Aldehyde	Increase	Kwak 2008 [66], Osada 2008 [65], Schaefer 2010 [25], Arnaiz 2014 [67]	Blood, Feces, Saliva
13	beta-Famesene	Hydrocarbon	Increase	Osada 2008 [65], Arnaiz 2014 [67]	Feces
14	alpha-Farnesene	Hydrocarbon	Increase	Kwak 2008 [66]	Feces
15	2,2,4-Trimethyl-1,3-pentanediol 1-monoisobutyrate (texanol)	Hydrocarbon	Increase	Kwak 2011 [68]	N.D.
16	2,2,4-Trimethyl-1,3-pentanediol 3-monoisobutyrate (texanol isomer)	Hydrocarbon	Increase	N.D.	N.D.
17	5,9-Undecadien-2-ol, 6,10-dimethyl-		Increase	N.D. (5,9-Undecadien-2-one 6,10-dimethyl- is found in mouse., Cavaggioni 2008 [69])	N.D.

^a)^ Numbers correspond to the numbers in Table 1.

^b)^ Values in KO mice relative to those in WT mice (fold) indicate decreases (< 1.0) or increases (< 1.0), from Table 1.

Furthermore, identified groups via PCA and hierarchical cluster analysis were divided by their functions. In group 1, the ‘six VOCs’ included pheromones: 5,9-undecadien-2-ol, 6,10-dimethyl and benzaldehyde are known as insect pheromones [59,60,61]; 3-heptanone 6-methyl, a derivative of 6-hydroxy-6-methyl-3-heptanone which is a known mouse pheromone [62]. The ‘six VOCs’ in group 1 were found to be correlated with the startle test. Alternatively, in group 2, mice pheromones classified as hydrocarbons were included, namely alpha-farnesene and beta-farnesene, which were altered in St3gal4-KO mice and following social behavior. Hence, St3gal4 affected the expression of pheromones that were dependent on differential behaviors. Lastly, in group 3, texanol and texanol isomer were observed, which are common plasticizers in water-based paints and certain plastic materials [63]. Hence, in young mice, social behaviors may have affected the metabolisms of exogenous compounds regardless of St3gal4-KO/WT. Taken together, the proposed metabolic pathways from the VOC chemical classes as well as the observed relationships between VOCs and emotions, suggest an association between metabolism and emotional behavior. Identification of the biological pathways of these metabolites may provide insights into the relationships between peripheral metabolites and psychiatric effects.

## Supporting information

S1 Fig Typical GC-MS total ion chromatograms of volatile organic compounds (VOCs) from the urine samples of St3gal4 WT and KO in experiment 2. TICs were obtained from analysis of the samples by HS-SPME and GC-MS, which were performed as described in Materials and methods. The TIC chromatograms are shown as relative intensity when the absolute intensity of peak no. 3 is 100%. Numbers indicate the following metabolites with similarity indexes of above 85%: 1) carbamic acid, monoammonium salt; 2) methylamine, *N*,*N*-dimethyl-; 3) 4-octen-3-one, 6-ethyl-7-hydroxy-; 4) 2-pentanone; 5) 2-propyl-1-pentanol; 6) 2-hexenal, 2-ethyl-; 7) ethanone, 1-cyclopropyl-; 8) 2-heptanone; 9) pentanoic acid, 4-methyl-, ethyl ester; 10) 3-heptanone, 6-methyl-; 11) 5-hexen-2-one, 5-methyl-; 12) 5-oxohexanenitrile; 13) 2,4,4-trimethyl-1-pentanol, trifluoroacetate; 14) pentane, 2-nitro-; 15) 3-heptanone, 5-methylene-; 16) 2-acetyl-1-pyrroline; 17) 2-pyrrolidinemethanol, 1-methyl-; 18) 7-exo-ethyl-5-methyl-6,8-dioxabicyclo[3.2.1]oct-3-ene; 19) benzaldehyde; 20) butanoic acid, 3-methyl-; 21) β-farnesene; 22) α-farnesene; 23) hexanoic acid; 24) 2,2,4-trimethyl, 1,3-pentandiol, 1-monoisobutylate (texanol); 25) dimethyl sulfone; 26) 5,9-undecadien-2-ol, 6,10-dimethyl-; 27) ethanol, 2,2'-oxybis-; 28) ethanone, 1-(1H-pyrrol-2-yl)-; 29) formamide, N-phenyl-; 30) eicosyl acetate.
(TIF)

S1 Table Volatile organic compound (VOC) names, similarity indexes, chemical formulas, CAS nos., and molecular weights, as analyzed by GC-MS using an InertCap PureWAX column under the operating parameters of experiment 1. *Compounds that are also shown in S2 Table are labeled. Operation parameters for the mass spectrometer in experiment 1 were described in the Materials and Methods.
(DOCX)

S2 Table VOC names, similarity indexes, chemical formulas, CAS nos., and molecular weights, as analyzed by GC-MS using an InertCap PureWAX column under the operating parameters of experiment 2. *Compounds that are also shown in S1 Table are labeled. Operation parameters for the mass spectrometer in experiment 2 are described in the Materials and Methods.
(DOCX)

S3 Table Significantly changed VOCs (*p* < 0.05) in the urine of St3gal4-KO and WT mice, as analyzed by GC-MS under the operating parameters of experiment 1 using XCMS. VOCs were obtained by XCMS analysis. Quantified ions were used to calculate the peak areas of VOC values and are represented as fold-changes of the average peak area of St3gal4-KO mice relative to that of WT mice. *VOCs commonly obtained in experiments 1 and 2.
(DOCX)

S4 Table Significantly changed VOCs (*p* < 0.05) in the urine of St3gal4-KO and WT mice, as analyzed by GC-MS under the operating parameters of experiment 2 by XCMS. VOCs were obtained by XCMS analysis. Quantified ions were used to calculate the peak areas of VOCs. Values are presented as fold-changes of the average peak area of St3gal4-KO mice relative to that of WT mice. *VOCs commonly obtained in experiments 1 and 2.
(DOCX)

S5 Table Statistical analysis of behavior-related VOCs in St3gal4-KO and WT mice by Kruskal-Wallis test. Following Kruskal-Wallis tests (A) two multiple comparison tests by Dunn’s test (B) and by controlling the false discovery rate (C) were performed using the absolute value of urinary VOCs shown as Table 2. Descriptive statistics are shown in (D).
(XLSX)
