# Supplementary figures and images for: Urinary volatilome analysis in a mouse model of anxiety and depression
Source: PLoS One. 2020 Feb 21;15(2):e0229269. doi: 10.1371/journal.pone.0229269 (PMC7034835; doi:10.1371/journal.pone.0229269)

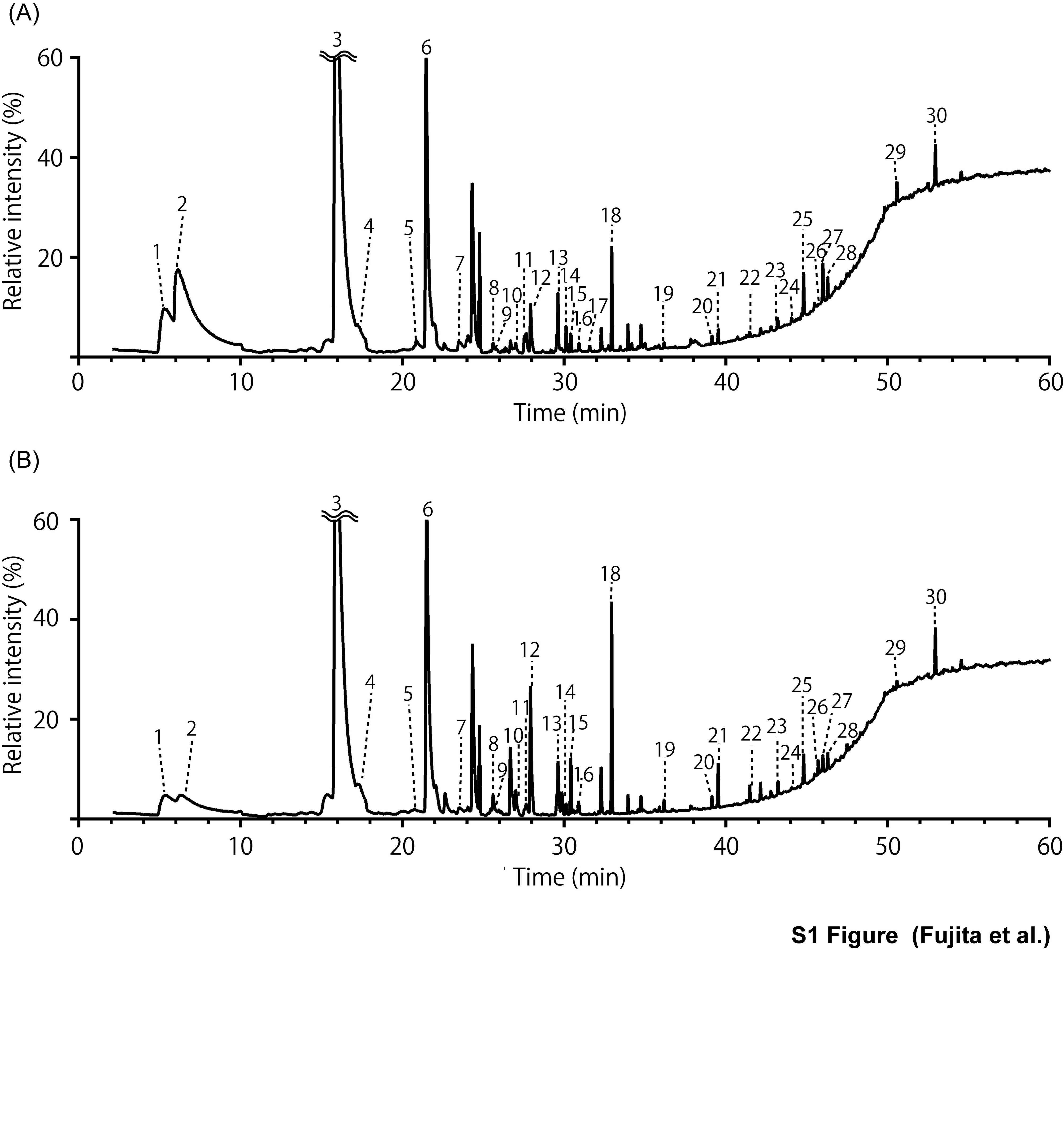

Supplement: S1 Fig — TICs were obtained from analysis of the samples by HS-SPME and GC-MS, which were performed as described in Materials and methods. The TIC chromatograms are shown as relative intensity when the absolute intensity of peak no. 3 is 100%. Numbers indicate the following metabolites with similarity indexes of above 85%: 1) carbamic acid, monoammonium salt; 2) methylamine, N,N-dimethyl-; 3) 4-octen-3-one, 6-ethyl-7-hydroxy-; 4) 2-pentanone; 5) 2-propyl-1-pentanol; 6) 2-hexenal, 2-ethyl-; 7) ethanone, 1-cyclopropyl-; 8) 2-heptanone; 9) pentanoic acid, 4-methyl-, ethyl ester; 10) 3-heptanone, 6-methyl-; 11) 5-hexen-2-one, 5-methyl-; 12) 5-oxohexanenitrile; 13) 2,4,4-trimethyl-1-pentanol, trifluoroacetate; 14) pentane, 2-nitro-; 15) 3-heptanone, 5-methylene-; 16) 2-acetyl-1-pyrroline; 17) 2-pyrrolidinemethanol, 1-methyl-; 18) 7-exo-ethyl-5-methyl-6,8-dioxabicyclo[3.2.1]oct-3-ene; 19) benzaldehyde; 20) butanoic acid, 3-methyl-; 21) β-farnesene; 22) α-farnesene; 23) hexanoic acid; 24) 2,2,4-trimethyl, 1,3-pentandiol, 1-monoisobutylate (texanol); 25) dimethyl sulfone; 26) 5,9-undecadien-2-ol, 6,10-dimethyl-; 27) ethanol, 2,2'-oxybis-; 28) ethanone, 1-(1H-pyrrol-2-yl)-; 29) formamide, N-phenyl-; 30) eicosyl acetate. (TIF) [file pone.0229269.s001.tif]
