# Supplementary material for: Urinary volatilome analysis in a mouse model of anxiety and depression
Source: PLoS One. 2020 Feb 21;15(2):e0229269. doi: 10.1371/journal.pone.0229269 (PMC7034835; doi:10.1371/journal.pone.0229269)
Supplement: S1 Table — *Compounds that are also shown in S2 Table are labeled. Operation parameters for the mass spectrometer in experiment 1 were described in the Materials and Methods. (DOCX) [file pone.0229269.s002.docx]

**S1 Table. Volatile organic compound (VOC) names, similarity indexes, chemical formulas, CAS nos., and molecular weights, as analyzed by GC-MS using an InertCap PureWAX column under the operating parameters of experiment 1.**

| No | RT | SI | VOCs | Chemical Formula | CAS | MW |
| --- | --- | --- | --- | --- | --- | --- |
| 1 | 8.885 | 94 | (2-Aziridinylethyl)amine | C_4_H_10_N_2_ | 4025-37-0 | 86 |
| 2 | 8.915 | 90 | Carbamic acid, monoammonium salt* | CH_6_N_2_O_2_ | 1111-78-0 | 78 |
| 3 | 11.983 | 96 | Methylamine, N,N-dimethyl-* | C_3_H_9_N | 75-50-3 | 59 |
| 4 | 12.262 | 85 | Tetramethylammonium perchlorate | C_4_H_12_ClNO_4_ | 2537-36-2 | 173 |
| 5 | 15.797 | 88 | Acetone | C_3_H_6_O | 67-64-1 | 58 |
| 6 | 19.215 | 85 | Butanal, 2-methyl-* | C_5_H_10_O | 96-17-3 | 86 |
| 7 | 19.382 | 86 | Butanal, 3-methyl- | C_5_H_10_O | 590-86-3 | 86 |
| 8 | 20.124 | 97 | Ethanol* | C_2_H_6_O | 64-17-5 | 46 |
| 9 | 20.57 | 86 | 4-Octen-3-one, 6-ethyl-7-hydroxy-* | C_10_H_18_O_2_ | 78464-96-7 | 170 |
| 10 | 21.841 | 91 | 2-Pentanone* | C_5_H_10_O | 107-87-9 | 86 |
| 11 | 25.113 | 85 | 1-Butanol, 2-methyl- | C_5_H_12_O | 137-32-6 | 88 |
| 12 | 26.731 | 86 | 2-Hexenal, 2-ethyl-* | C_8_H_14_O | 645-62-5 | 126 |
| 13 | 27.53 | 86 | Ethanone, 1-cyclopropyl-* | C_5_H_8_O | 765-43-5 | 84 |
| 14 | 27.56 | 86 | 3-Penten-2-one | C_5_H_8_O | 625-33-2 | 84 |
| 15 | 27.715 | 90 | Ethylbenzene | C_8_H_10_ | 100-41-4 | 106 |
| 16 | 27.778 | 92 | 1-Butanol | C_4_H_10_O | 71-36-3 | 74 |
| 17 | 29.507 | 94 | 2-Heptanone* | C_7_H_14_O | 110-43-0 | 114 |
| 18 | 30.082 | 90 | 2-Butenal, 3-methyl- | C_5_H_8_O | 107-86-8 | 84 |
| 19 | 30.573 | 94 | 3-Heptanone, 6-methyl-* | C_8_H_16_O | 624-42-0 | 128 |
| 20 | 30.812 | 93 | 2-Penten-1-ol, acetate, (Z)-* | C_7_H_12_O_2_ | 42125-10-0 | 128 |
| 21 | 30.835 | 87 | Cyclopentanone, 3-methyl-* | C_6_H_10_O | 1757-42-2 | 98 |
| 22 | 31.733 | 89 | 5-Oxohexanenitrile* | C_6_H_9_NO | 10412-98-3 | 111 |
| 23 | 31.74 | 89 | 5-Hexen-2-one, 5-methyl-* | C_7_H_12_O | 3240-09-3 | 112 |
| 24 | 31.767 | 89 | 5-Hepten-2-one | C_7_H_12_O | 6714-00-7 | 112 |
| 25 | 32.147 | 90 | Styrene | C_8_H_8_ | 100-42-5 | 104 |
| 26 | 33.31 | 89 | 3-Heptanone, 5-methylene-* | C_8_H_14_O | 20690-70-4 | 126 |
| 27 | 33.318 | 89 | 6-Hepten-3-one, 4-methyl- | C_8_H_14_O | 26118-97-8 | 126 |
| 28 | 33.409 | 90 | 3-Hepten-2-one | C_7_H_12_O | 1119-44-4 | 112 |
| 29 | 33.94 | 93 | Pentane, 2-nitro-* | C_5_H_11_NO_2_ | 4609-89-6 | 117 |
| 30 | 34.44 | 88 | 5-Hepten-2-one, 6-methyl- | C_8_H_14_O | 110-93-0 | 126 |
| 31 | 36.532 | 85 | Nonadecane | C_19_H_40_ | 629-92-5 | 268 |
| 32 | 40.039 | 96 | Benzaldehyde* | C_7_H_6_O | 100-52-7 | 106 |
| 33 | 43.203 | 94 | beta-Famesene* | C_15_H_24_ | 18794-84-8 | 204 |
| 34 | 45.16 | 96 | alpha-Farnesene* | C_15_H_24_ | 502-61-4 | 204 |
| 35 | 47.789 | 94 | Propanoic acid, 2-methyl-, 3-hydroxy-2,2,4-trimethylpentyl ester* | C_12_H_24_O_3_ | 77-68-9 | 216 |
| 36 | 48.227 | 94 | 2,2,4-Trimethyl-1,3-pentanediol diisobutyrate | C_16_H_30_O_4_ | 6846-50-0 | 286 |
| 37 | 49.35 | 91 | 5,9-Undecadien-2-ol, 6,10-dimethyl- | C_13_H_24_O | 53837-34-6 | 196 |
| 38 | 52.176 | 96 | p-Cresol | C_7_H_8_O | 106-44-5 | 108 |
| 39 | 53.187 | 87 | 5-Tetradecen-1-ol, acetate, (Z)- | C_16_H_30_O_2_ | 35153-13-0 | 254 |
| 40 | 57.774 | 93 | 1-Docosanol, acetate | C_24_H_48_O_2_ | 822-26-4 | 368 |
| 41 | 59.191 | 85 | 2,6,10-Dodecatrien-1-ol, 3,7,11-trimethyl- | C_15_H_26_O | 4602-84-0 | 222 |
| 42 | 59.855 | 88 | n-Nonadecanol-1 | C_19_H_40_O | 1454-84-8 | 284 |

*Compounds that are also shown in S2 Table are labeled. Operation parameters for the mass spectrometer in experiment 1 were described in the Materials and Methods.
