# Supplementary material for: Urinary volatilome analysis in a mouse model of anxiety and depression
Source: PLoS One. 2020 Feb 21;15(2):e0229269. doi: 10.1371/journal.pone.0229269 (PMC7034835; doi:10.1371/journal.pone.0229269)
Supplement: S2 Table — *Compounds that are also shown in S1 Table are labeled. Operation parameters for the mass spectrometer in experiment 2 are described in the Materials and Methods. (DOCX) [file pone.0229269.s003.docx]

**S2 Table. VOC names, similarity indexes, chemical formulas, CAS nos., and molecular weights, as analyzed by GC-MS using an InertCap PureWAX column under the operating parameters of experiment 2.**

| No | RT (min) | SI | VOCs | Chemical Formula | CAS | MW |
| --- | --- | --- | --- | --- | --- | --- |
| 1 | 5.409 | 96 | Carbamic acid, monoammonium salt* | CH_6_N_2_O_2_ | 1111-78-0 | 78 |
| 2 | 5.418 | 96 | Carbon dioxide | CO_2_ | 124-38-9 | 44 |
| 3 | 6.178 | 97 | Methylamine, N,N-dimethyl-* | C_3_H_9_N | 75-50-3 | 59 |
| 4 | 10.002 | 85 | Hydroxyurea | CH_4_N_2_O_2_ | 127-07-1 | 76 |
| 5 | 13.727 | 95 | 2-Butanone | C_4_H_8_O | 78-93-3 | 72 |
| 6 | 14.308 | 93 | Butanal, 2-methyl-* | C_5_H_10_O | 96-17-3 | 86 |
| 7 | 15.342 | 96 | Ethanol* | C_2_H_6_O | 64-17-5 | 46 |
| 8 | 15.884 | 85 | 4-Octen-3-one, 6-ethyl-7-hydroxy-* | C_10_H_18_O_2_ | 78464-96-7 | 170 |
| 9 | 17.217 | 95 | 2-Pentanone* | C_5_H_10_O | 107-87-9 | 86 |
| 10 | 17.22 | 86 | Ethanone, 1-oxiranyl- | C_4_H_6_O_2_ | 4401-11-0 | 86 |
| 11 | 19.013 | 90 | 2-Pentanone, 3-methyl- | C_6_H_12_O | 565-61-7 | 100 |
| 12 | 20.533 | 88 | 3-Hexanone | C_6_H_12_O | 589-38-8 | 100 |
| 13 | 20.739 | 88 | 2,3-Pentanedione | C_5_H_8_O_2_ | 600-14-6 | 100 |
| 14 | 20.866 | 90 | Heptane, 2,5-dimethyl- | C_9_H_20_ | 2216-30-0 | 128 |
| 15 | 20.868 | 91 | 2-Propyl-1-pentanol | C_8_H_18_O | 58175-57-8 | 130 |
| 16 | 20.892 | 89 | 3-Pentanone, 2-methyl- | C_6_H_12_O | 565-69-5 | 100 |
| 17 | 20.899 | 89 | Tert-butyl[(cyanomethyl)-oxo- | C_6_H_11_N_3_O | 0-00-0 | 141 |
| 18 | 21.502 | 86 | 2-Hexenal, 2-ethyl-* | C_8_H_14_O | 645-62-5 | 126 |
| 19 | 23.484 | 92 | Ethanone, 1-cyclopropyl-* | C_5_H_8_O | 765-43-5 | 84 |
| 20 | 23.528 | 88 | 3-Hexanone, 2-methyl- | C_7_H_14_O | 7379-12-6 | 114 |
| 21 | 25.609 | 94 | 2-Heptanone* | C_7_H_14_O | 110-43-0 | 114 |
| 22 | 25.614 | 92 | 2-Hexanone, 5-methyl- | C_7_H_14_O | 110-12-3 | 114 |
| 23 | 25.817 | 91 | p-Xylene | C_8_H_10_ | 106-42-3 | 106 |
| 24 | 25.845 | 88 | Benzene, 1,3-dimethyl- | C_8_H_10_ | 108-38-3 | 106 |
| 25 | 25.999 | 88 | Pentanoic acid, 4-methyl-, ethyl ester | C_8_H_16_O_2_ | 25415-67-2 | 144 |
| 26 | 26.182 | 94 | 1-Tetrazol-2-ylethanone | C_3_H_4_N_4_O | 51410-11-8 | 112 |
| 27 | 26.445 | 88 | D-Limonene | C_10_H_16_ | 5989-27-5 | 136 |
| 28 | 26.697 | 94 | 3-Heptanone, 6-methyl-* | C_8_H_16_O | 624-42-0 | 128 |
| 29 | 26.846 | 95 | (R)-(+)-3-Methylcyclopentanone | C_6_H_10_O | 6672-30-6 | 98 |
| 30 | 26.852 | 89 | Cyclopentanone, 3-methyl-* | C_6_H_10_O | 1757-42-2 | 98 |
| 31 | 27.013 | 96 | 2-Penten-1-ol, acetate, (Z)-* | C_7_H_12_O_2_ | 42125-10-0 | 128 |
| 32 | 27.655 | 88 | 4-Hepten-2-one, (E)- | C_7_H_12_O | 36678-43-0 | 112 |
| 33 | 27.667 | 85 | 5-Hexen-2-one, 5-methyl-* | C_7_H_12_O | 3240-09-3 | 112 |
| 34 | 27.931 | 90 | 5-Oxohexanenitrile* | C_6_H_9_NO | 10412-98-3 | 111 |
| 35 | 29.198 | 91 | Acetoin | C_4_H_8_O_2_ | 513-86-0 | 88 |
| 36 | 29.439 | 90 | 2,4-Dithiapentane | C_3_H_8_S_2_ | 1618-26-4 | 108 |
| 37 | 30.127 | 93 | Pentane, 2-nitro-* | C_5_H_11_NO_2_ | 4609-89-6 | 117 |
| 38 | 30.419 | 90 | 3-Heptanone, 5-methylene-* | C_8_H_14_O | 20690-70-4 | 126 |
| 39 | 30.9 | 86 | 2-Acetyl-1-pyrroline | C_6_H_9_NO | 85213-22-5 | 111 |
| 40 | 31.557 | 91 | 2-Pyrrolidinemethanol, 1-methyl- | C_6_H_13_NO | 3554-65-2 | 115 |
| 41 | 32.759 | 87 | 1-Nitro-2-methyl propene | C_4_H_7_NO_2_ | 1606-30-0 | 101 |
| 42 | 32.943 | 88 | 7-Exo-ethyl-5-methyl-6,8-dioxabicyclo[3.2.1]oct-3-ene | C_9_H_14_O_2_ | 62255-25-8 | 154 |
| 43 | 33.466 | 97 | Acetic acid | C_2_H_4_O_2_ | 64-19-7 | 60 |
| 44 | 33.793 | 92 | 2-Butene, 1-bromo-3-methyl- | C_5_H_9_Br | 870-63-3 | 148 |
| 45 | 34.171 | 85 | Oxetane, 2-methyl-4-propyl- | C_7_H_14_O | 7045-79-6 | 114 |
| 46 | 34.176 | 87 | Hydroperoxide, 1-methylpentyl | C_6_H_14_O_2_ | 24254-55-5 | 118 |
| 47 | 34.181 | 86 | Hydroperoxide, 1-methylhexyl | C_7_H_16_O_2_ | 762-46-9 | 132 |
| 48 | 35.014 | 85 | Diallyl carbonate | C_7_H_10_O_3_ | 15022-08-9 | 142 |
| 49 | 35.796 | 94 | Propanoic acid | C_3_H_6_O_2_ | 79-09-4 | 74 |
| 50 | 36.179 | 97 | Benzaldehyde* | C_7_H_6_O | 100-52-7 | 106 |
| 51 | 36.585 | 91 | Propanoic acid, 2-methyl- | C_4_H_8_O_2_ | 79-31-2 | 88 |
| 52 | 36.705 | 94 | 2,3-Butanediol, [R-(R*,R*)]- | C_4_H_10_O_2_ | 24347-58-8 | 90 |
| 53 | 36.857 | 86 | Propanoic acid, 2,2-dimethyl- | C_5_H_10_O_2_ | 75-98-9 | 102 |
| 54 | 38.088 | 91 | Butanoic acid | C_4_H_8_O_2_ | 107-92-6 | 88 |
| 55 | 39.141 | 97 | Butanoic acid, 3-methyl- | C_5_H_10_O_2_ | 503-74-2 | 102 |
| 56 | 39.529 | 94 | beta-Famesene* | C_15_H_24_ | 18794-84-8 | 204 |
| 57 | 40.734 | 95 | Pentanoic acid | C_5_H_10_O_2_ | 109-52-4 | 102 |
| 58 | 41.481 | 97 | alpha-Farnesene* | C_15_H_24_ | 502-61-4 | 204 |
| 59 | 42.786 | 93 | Benzenemethanol, .alpha.-methyl- | C_8_H_10_O | 98-85-1 | 122 |
| 60 | 42.788 | 89 | o-Toluidine | C_7_H_9_N | 95-53-4 | 107 |
| 61 | 42.797 | 88 | Benzenamine, 3-methyl- | C_7_H_9_N | 108-44-1 | 107 |
| 62 | 43.19 | 96 | Hexanoic acid | C_6_H_12_O_2_ | 142-62-1 | 116 |
| 63 | 44.064 | 91 | Propanoic acid, 2-methyl-, 3-hydroxy-2,2,4-trimethylpentyl ester* | C_12_H_24_O_3_ | 77-68-9 | 216 |
| 64 | 44.811 | 96 | Dimethyl sulfone | C_2_H_6_O_2_S | 67-71-0 | 94 |
| 65 | 45.478 | 95 | Hexanoic acid, 2-ethyl- | C_8_H_16_O_2_ | 149-57-5 | 144 |
| 66 | 45.713 | 92 | 5,9-Undecadien-2-ol, 6,10-dimethyl- | C_13_H_24_O | 53837-34-6 | 196 |
| 67 | 45.995 | 98 | Ethanol, 2,2'-oxybis- | C_4_H_10_O_3_ | 111-46-6 | 106 |
| 68 | 46.297 | 96 | Ethanone, 1-(1H-pyrrol-2-yl)- | C_6_H_7_NO | 1072-83-9 | 109 |
| 69 | 47.71 | 86 | Octanoic acid | C_8_H_16_O_2_ | 124-07-2 | 144 |
| 70 | 50.574 | 91 | Formamide, N-phenyl- | C_7_H_7_NO | 103-70-8 | 121 |
| 71 | 52.953 | 95 | Eicosyl acetate | C_22_H_44_O_2_ | 0-00-0 | 340 |
| 72 | 54.556 | 90 | n-Pentadecanol | C_15_H_32_O | 629-76-5 | 228 |
| 73 | 54.557 | 86 | 1-Tetradecanol | C_14_H_30_O | 112-72-1 | 214 |
| 74 | 54.568 | 95 | 1-Hexadecanol | C_16_H_34_O | 36653-82-4 | 242 |
| 75 | 54.572 | 91 | Eicosyl methyl ether | C_21_H_44_O | 0-00-0 | 312 |

*Compounds that are also shown in S1 Table are labeled. Operation parameters for the mass spectrometer in experiment 2 are described in the Materials and Methods.
