# Supplementary material for: Urinary volatilome analysis in a mouse model of anxiety and depression
Source: PLoS One. 2020 Feb 21;15(2):e0229269. doi: 10.1371/journal.pone.0229269 (PMC7034835; doi:10.1371/journal.pone.0229269)
Supplement: S3 Table — VOCs were obtained by XCMS analysis. Quantified ions were used to calculate the peak areas of VOC values and are represented as fold-changes of the average peak area of St3gal4-KO mice relative to that of WT mice. *VOCs commonly obtained in experiments 1 and 2. (DOCX) [file pone.0229269.s004.docx]

**S3 Table. Significantly changed VOCs (p < 0.05) in the urine of St3gal4-KO and WT mice, as analyzed by GC-MS under the operating parameters of experiment 1 using XCMS.**

| VOC | Observed *m/z* | Retention time (min) | Quantified ion (*m/z*) | Fold-change | *p* value |
| --- | --- | --- | --- | --- | --- |
| 1* | 99 , 57 , 81 , 128 , 72 | 30.567 | 57 | 7.713 | 0.0001 |
| 2 | 68 , 112 , 37 , 38 , 94 , 43 , 41 | 31.617 | 43 | 3.066 | 0.0020 |
| 3 | 50 , 52 , 51 , 104 , 78 , 63 | 32.150 | 104 | 2.250 | 0.0269 |
| 4 | 55 | 33.417 | 55 | 1.587 | 0.0035 |
| 5* | 57 , 41 , 97 , 69 | 34.150 | 57 | 5.841 | 0.0002 |
| 6* | 106 , 105 , 77 , 50 , 51 | 40.033 | 106 | 3.233 | 0.0000 |
| 7* | 93 , 120 , 92 , 69 , 79 , 81 , 94 | 43.217 | 69 | 3.638 | 0.0057 |
| 8* | 93 , 123 , 107 | 45.167 | 93 | 4.974 | 0.0052 |
| 9 | 71 , 143 , 173 , 56 | 47.767 | 71 | 1.309 | 0.0193 |
| 10 | 83, 98, 143 | 48.450 | 83 | 1.302 | 0.0159 |
| 11 | 153, 155 | 49.350 | 153 | 4.393 | 0.0068 |
| 12 | 83, 196, 82, 111, 125, 98, 61, 96, 84, 139 | 58.85 | 83 | 1.799 | 0.0017 |

VOCs were obtained by XCMS analysis. Quantified ions were used to calculate the peak areas of VOC values and are represented as fold-changes of the average peak area of St3gal4-KO mice relative to that of WT mice. *VOCs commonly obtained in experiments 1 and 2.
