# Supplementary material for: Urinary volatilome analysis in a mouse model of anxiety and depression
Source: PLoS One. 2020 Feb 21;15(2):e0229269. doi: 10.1371/journal.pone.0229269 (PMC7034835; doi:10.1371/journal.pone.0229269)
Supplement: S4 Table — VOCs were obtained by XCMS analysis. Quantified ions were used to calculate the peak areas of VOCs. Values are presented as fold-changes of the average peak area of St3gal4-KO mice relative to that of WT mice. *VOCs commonly obtained in experiments 1 and 2. (DOCX) [file pone.0229269.s005.docx]

**S4 Table.** **Significantly changed VOCs (*p* < 0.05) in the urine of St3gal4-KO and WT mice, as analyzed by GC-MS under the operating parameters of experiment 2 by XCMS.**

| No. | Observed *m/z* | Retention time (min) | Quantified ion (*m/z*) | Fold-change | *p* value |
| --- | --- | --- | --- | --- | --- |
| 1 | 56 , 59 , 42 , 57 , 58 | 6.183 | 58 | 0.408 | 0.0003 |
| 2 | 64 , 62 , 63 , 58 , 110 , 50 , 38 , 95 , 96 , 54 , 81 , 51 , 82 , 65 , 53 , 40 , 84 , 93 , 52 , 66 , 41 , 77 , 91 , 111 , 125 , 55 , 83 , 79 , 69 , 97 , 67 , 78 , 127 , 92 , 85 , 68 , 56 , 126 , 128 , 57 , 80 , 98 , 70 , 107 , 112 , 94 , 59 , 108 , 109 , 42 , 39 , 37 , 43 , 49 | 15.967 | 57 | 1.478 | 0.0408 |
| 3 | 85, 111 | 22.717 | 111 | 2.165 | <0.0001 |
| 4 | 37 , 38 , 84 , 69 | 23.550 | 69 | 1.685 | 0.0010 |
| 5 | 106, 91 | 25.883 | 106 | 1.689 | 0.0103 |
| 6 | 72 , 99 , 128 , 81 | 26.700 | 81 | 2.584 | 0.0299 |
| 7* | 94 , 43 , 138 | 27.950 | 43 | 1.762 | 0.0428 |
| 8* | 57 | 30.433 | 57 | 2.376 | 0.0037 |
| 9* | 106 , 77 , 50 , 51 , 105 | 36.183 | 106 | 1.461 | 0.0094 |
| 10* | 204 , 107 , 162 , 92 , 91 , 79 , 93 , 161 | 39.533 | 93 | 3.905 | 0.0296 |
| 11* | 107 , 119 , 123 | 41.483 | 107 | 2.046 | 0.0276 |

VOCs were obtained by XCMS analysis. Quantified ions were used to calculate the peak areas of VOCs. Values are presented as fold-changes of the average peak area of St3gal4-KO mice relative to that of WT mice. *VOCs commonly obtained in experiments 1 and 2.
